# Major Role of Voluminosity in the Compressibility and Sol–Gel Transition of Casein Micelle Dispersions Concentrated at 7 °C and 20 °C

**DOI:** 10.3390/foods8120652

**Published:** 2019-12-06

**Authors:** Floriane Doudiès, Anne-Sophie Arsène, Fabienne Garnier-Lambrouin, Marie-Hélène Famelart, Antoine Bouchoux, Frédéric Pignon, Geneviève Gésan-Guiziou

**Affiliations:** 1 STLO, INRA, AGROCAMPUS OUEST, 35000 Rennes, France; floriane.doudies@inra.fr (F.D.); anne-so.arsene@outlook.fr (A.-S.A.); fabienne.lambrouin@inra.fr (F.G.-L.); marie-helene.famelart@inra.fr (M.-H.F.); 2 TBI, Université de Toulouse, CNRS, INRA, INSA, 31000 Toulouse, France; antoine.bouchoux@insa-toulouse.fr; 3 University Grenoble Alpes, CNRS, Grenoble INP (Institute of Engineering University Grenoble Alpes), LRP, F-38000 Grenoble, France; frederic.pignon@univ-grenoble-alpes.fr

**Keywords:** casein micelle, osmotic pressure, sol–gel transition, rheology, temperature

## Abstract

The objective of this work is to bring new information about the influence of temperatures (7 °C and 20 °C) on the equation of state and sol–gel transition behavior of casein micelle dispersions. Casein micelle dispersions have been concentrated and equilibrated at different osmotic pressures using equilibrium dialysis at 7 °C and 20 °C. The osmotic stress technique measured the osmotic pressures of the dispersions over a wide range of concentrations. Rheological properties of concentrated dispersions were then characterized, respectively at 7 °C and at 20 °C. The essential result is that casein micelle dispersions are less compressible at 7 °C than at 20 °C and that concentration of sol–gel transition is lower at 7 °C than at 20 °C, with compressibility defined as the inverse to the resistance to the compression, and that is proportional to the cost to remove water from structure. From our interpretations, these two features were fully consistent with a release of soluble β-casein and nanoclusters CaP and an increased casein micelle hydration and apparent voluminosity at 7 °C as compared with 20 °C.

## 1. Introduction

Casein micelles are colloidal particles that account for 80% of the total protein content of cow milk. They are largely involved in the dairy industry, for example, in cheese and yogurt manufacturing, and play an important role in milk processing, especially in concentration operations. Casein micelles are large assemblies composed of four different caseins (*α_s_*_1_, *α_s_*_2_, *β,* and *κ* in proportion of 4:1:4:1) and 8 wt. % of phosphate and calcium ions [1]. The structure of casein micelles is still controversial, but it is commonly accepted that they have a roughly spherical shape and a core–shell structure, with the outer diameter ranging from 50 to 500 nm [2,3,4,5]. The core is generally described as a matrix of proteins in which the ionic nanoclusters of calcium and phosphate, randomly distributed, act as connecting points [6,7,8,9,10]. There is a consensus on the casein micelle shell that is essentially made of *κ*-caseins protruding into the aqueous phase as a polyelectrolyte brush and stabilizing casein micelles through electrostatic and steric repulsions and insuring its hydration [11,12]. Moreover, casein micelles contain 76 wt. % of water. They look like some kind of natural sponge-like microgels that are porous, deformable, compressible, and dynamic [13].

Most of the important mass exchange operations including milk filtration, evaporation, and drying involve concentrating casein micelles. The efficiency of these operations is then strongly dependent on colloidal compressibility and permeability and on rheological properties of concentrated dispersions of casein micelles. Therefore, the behavior of casein micelles in concentrated dispersions has been extensively studied over the last years. The equation of state (relationship between concentration and applied osmotic pressure during an osmotic stress experiment) of casein micelles was obtained at 20 °C for the wide range of casein concentrations from 20 to 800 g/L [14,15]. The rheological behavior of casein micelles was also investigated at 20–25 °C [16,17,18,19,20]. Both approaches have shown that casein micelle dispersions behave more and more like a solid (sol–gel transition) when their volume fraction reaches a critical value. It was found that at 20–25 °C, the sol–gel transition is in the range of effective particle volume fractions of 0.68–0.8 [16,17,18,19,20], with a specific concentration of sol–gel transition of 178 and 185 g/L respectively for Bouchoux et al. [16] and Jin et al. [18]. Previous works described three compression regimes and rheological behaviors at 20 °C [14,16]:

(1) a dilute regime, where casein micelles do not interact and behave as hard spheres;
(2) a sol–gel transition regime, where interactions between casein micelles become stronger and rheological behavior changes from liquid-like (sol) to solid-like (gel);
(3) a concentrated regime, where dispersions behave as a coherent solid made of directly contacting de-swelled and deformed casein micelles.

As it follows from the literature review, concentrated dispersions of casein micelles were predominantly characterized at the temperature of 20–25 °C, but there is an increasing interest in low-temperature (7–12 °C) milk filtration because of the increasing use of polymeric spiral membranes that are less expensive than ceramic ones. Due to their complex geometry, these polymeric membranes are preferably used at low temperature to limit bacterial growth.

It is well known that lowering the temperature induces structural and composition changes in casein micelles such as: (i) the dissociation of macromolecular assemblies by weakening hydrophobic interactions; (ii) the release of *β*-casein and of colloidal calcium and phosphate in the serum phase [9,21,22,23,24,25]; (iii) the increase of hydration and apparent voluminosity of casein micelles [20,24]. Rheological measurements of concentrated casein micelle dispersions were carried out at temperatures from 5 to 35 °C [20], but the casein concentration investigated was lower than 200 g/L, which is far below the range of concentrations that could be encountered at the membrane surface, for example, as already suggested and measured by small-angle X-ray scattering (SAXS) experiments and predicted in milk filtration [10,18,26,27].

To the best of the authors’ knowledge, physical and rheological properties of concentrated casein micelle dispersions have not been studied in detail at low temperature. In this work, properties at 7 °C were measured and compared with that at 20 °C in regimes from semi-diluted to highly concentrated in order to better understand casein micelle deposit properties during concentration processes. Two approaches were applied: (i) a thermodynamic approach in order to characterize the equation of state by measuring the osmotic pressure of casein micelles, and (ii) a rheological approach in order to characterize the sol–gel transition. The aim of this work was to answer the two following questions:

(1) How does the equation of state of casein micelle dispersions change with the equilibrium temperature at 20 °C or 7 °C?
(2) How does the sol–gel transition behavior of casein micelle dispersions change with the equilibrium temperature at 20 °C or 7 °C?

## 2. Materials and Methods

### 2.1. Casein Micelle Dispersion Preparation

Experiments were done with casein isolate powder, that is, a milk fraction enriched in micellar casein (Promilk 852B, Ingredia, Arras, France) dispersed in a permeate obtained by ultrafiltration (UF) of skim milk (UF permeate). To prepare casein micelle powder, diafiltration steps are used, and some authors showed that depending on the number of diafiltration done, casein micelle structure and properties can be modified, but this type of casein isolate powder has already been used and demonstrated good recovery of casein micelle properties when dispersed in UF permeate [13,14,15,16,26,28,29]. Therefore, in dispersions, it can be considered as a substrate model of milk. The casein isolate powder was obtained from a pasteurized skim milk by microfiltration with a 0.1 µm membrane and analyzed according to standard methods. It was supposed that the pasteurization was done at low time and temperature, as high temperature conditions would result in high association of whey proteins with casein micelle and subsequent detrimental impact on microfiltration performance.

The average composition of the casein isolate powder is given in Table 1.

The average total, non-casein and non-protein nitrogen matter contents in the powder were determined through the Kjeldahl method [30,31] using 6.38, 6.25, and 6.19 as respective converting factors. The casein content was determined as the difference between the total nitrogen matter content and the non-casein nitrogen matter content. Casein represents more than 75% of the powder total solids. The serum protein content was determined as a difference between the non-casein nitrogen matter content and the non-protein nitrogen matter content. The non-protein nitrogen fraction contains most of the small peptides (i.e., molecular mass < 10 kDa) present in solution. The mineral content was determined as the ash content by drying the powder to 550 °C during 5 h [32].

The lactose (4 wt. %) and fat (1.5 wt. %) content, expressed on the total dry solid basis, were determined by the powder manufacturer:

The UF permeate was prepared by ultrafiltration of a fresh skim milk at 12 °C (membrane 6338 HFK 328, cut-off of 5 kDa, Koch Membranes Systems, Lyon, France). Caseins and whey proteins were eliminated through this operation. The ionic composition of UF permeate was 3.3 mM Mg^2+^, 18.0 mM Na^+^, 42.7 mM K^+^, 7.5 mM Ca^2+^, 29.0 mM Cl^−^, 7.5 mM citrate, and 9.9 mM phosphate (all values are given as averages with ± 5% standard deviations) (see [33] for a full description of methods). Cations (Ca, Na, Mg, K) were determined by an atomic absorption spectrophotometer (SpectrAA 220FS, Agilent Technologie, Les Ulis, France), and anions (inorganic phosphates, citrates and chlorides) by chromatography method (Dionex ICS 3000 HPLC, Thermo Fischer Scientific, Villebon Courtaboeuf, France). Thiomersal and sodium azide, both purchased from Sigma-Aldrich (ST. Louis, MO, USA), were added to the UF permeate as preservatives at 0.02 wt. % and 0.05 wt. %, respectively.

The casein micelle dispersions were prepared by thoroughly mixing the casein isolate powder with UF permeate at 35 °C for 15 h, as previously suggested [28]. The dynamic light scattering analysis of casein micelle dispersions confirmed that casein micelle dispersions had a particle size distribution from 50 to 500 nm with an average diameter of 130 nm that is similar to that of native casein micelles. The pH of dispersions was 6.7 ± 0.1 at 20 °C, that is, the pH value of a fresh skim milk.

### 2.2. Osmotic Stress Experiments

The osmotic stress technique is based on water exchange between casein micelle dispersions in a dialysis bag and a reservoir of known osmotic pressure made of polyethylene glycol (PEG) solutions prepared in UF permeate [14,15,19,34]. After pressure equilibrium was reached between the bag and the reservoir, the water content in the dispersion was measured and the relation between osmotic pressure and casein concentration was obtained.

#### 2.2.1. Stressing Solutions

The stressing solutions were prepared by diluting PEG in UF permeate, allowing to maintain ion chemical potentials in both sides of dialysis bags. Two PEGs were used, both purchased from Sigma-Aldrich (St. Louis, MO, USA). A PEG with molecular weight of 35 kDa was used for the osmotic stress experiments at pressures under 500 kPa. According to Bouchoux et al. [14], 500 kPa corresponds to a casein concentration of 500 g/L at 20 °C. For osmotic pressures higher than 500 kPa, PEG 35 kDa could not be used because of its low solubility: 500 kPa corresponds to a concentration of 20 wt. % of PEG 35 kDa, which is almost the saturation concentration. Therefore, PEG 20 kDa was chosen for stressing solutions at osmotic pressure above 500 kPa as its solubility goes up to 50 wt. %. Stressing solution volume was much higher (1 L) in comparison with volume in dialysis bag (0.01 L) in order to avoid great difference of distribution of UF permeate compounds when PEG concentration increases.

#### 2.2.2. Theoretical Development for Osmotic Pressure Determination

Currently, two different equations that relate osmotic pressure of PEG solution with PEG concentration were presented in the literature.

Firstly, in several works [13,14,15,16,26,34], authors used Equation (1) in order to calculate osmotic pressure by using only PEG concentration:

(1) $$\log\left(\pi \right)=a+b\;{\left[PEG\right]}^{c}$$

where π is the osmotic pressure (Pa), [PEG] is PEG concentration (wt. %), and fitting parameters are equal to:

*a* = 0.49, *b* = 2.50, *c* = 0.24 for PEG 35 kDa at 20 °C [14],
*a* = 0.57, *b* = 2.75, *c* = 0.21 for PEG 20 kDa at 20 °C [34].

Unfortunately, this equation applies only at 20 °C.

Secondly, an equation of state for PEGs with different molecular weights was proposed by Cohen et al. [35] to determine the osmotic pressure π (Pa):

(2) $$\pi N^{9/5}=\pi {\left(\frac{M}{M_{m}\times 1000}\right)}^{9/5}=\;\frac{RT}{M_{m}\bar{V}}\left[\left(\frac{C}{C_{N}^{*}}\right)+\alpha {\left(\frac{C}{C_{N}^{*}}\right)}^{\frac{9}{4}}\right]$$

where *N* is the number of monomers per polymer chain, *M* is the molar mass of PEG (Da), *M_m_* is the monomer molecular weight of PEG (g/mol), R is the universal gas constant (J∙mol^−1^∙K^−1^), T is the temperature (K), $\bar{V}$ is the polymer partial specific volume (m^3^/g), *C* is the PEG concentration (g/m^3^), *α* is the crossover index (fitting parameter), and $C_{N}^{*}$ is the characteristic *N*-dependent polymer concentration. All chemical specificities appear in only one parameter, the crossover index *α*, which is related to a number of physical properties: microscopic structure, Flory radius, monomer size and volume, and strength and range of interactions. The parameter $C_{N}^{*}$ is associated with the crossover between dilute and semidilute regimes. It is defined as $C_{N}^{*}\sim N^{-4/5}{\bar{V}}^{-1}$ and corresponds to a semiquantitatively defined polymer overlap concentration [36,37]. According to Cohen et al. [35], $C_{N}^{*}$ is defined as the concentration where $\pi_{vH}=\pi_{dC}$ (with $\pi_{vH}$ and $\pi_{dC}$ the values of osmotic pressure calculated from the van’t Hoff’s law and des Cloizeaux [38] osmotic pressure expression, respectively), and therefore, $C_{N}^{*}=\alpha^{-4/5}$. When plotting $\pi N^{9/5}$ versus $C/C_{N}^{*}$, experimental data for PEG of any molecular weights from 300 Da to 20 kDa collapse to a single master curve. This gives *α* = 0.490 for $\bar{V}=0.825$ mL/g and $M_{m}=44$ g/mol at 20 °C for PEG of 300 to 20 kDa.

Contrary to the study of Cohen et al. [35], recent publications [39,40] have shown that there is a systematic dependence of *α* on the polymer chain length *N* and that *α* decreases monotonically toward an asymptotic value $\alpha^{*}$:

(3) $$\alpha \left(N\right)=\;\alpha^{*}\left[1+bN^{-p}\right]$$

where $\alpha^{*}$ = 0.43 ± 0.02, *b* = 5.3 ± 1.4, and *p* = 0.84 ± 1.5. According to Equation (3), the calculated values of *α* = 0.434 and *α* = 0.432 are obtained at 20 °C for 20 kDa and 35 kDa PEG, respectively.

**Figure 1:**
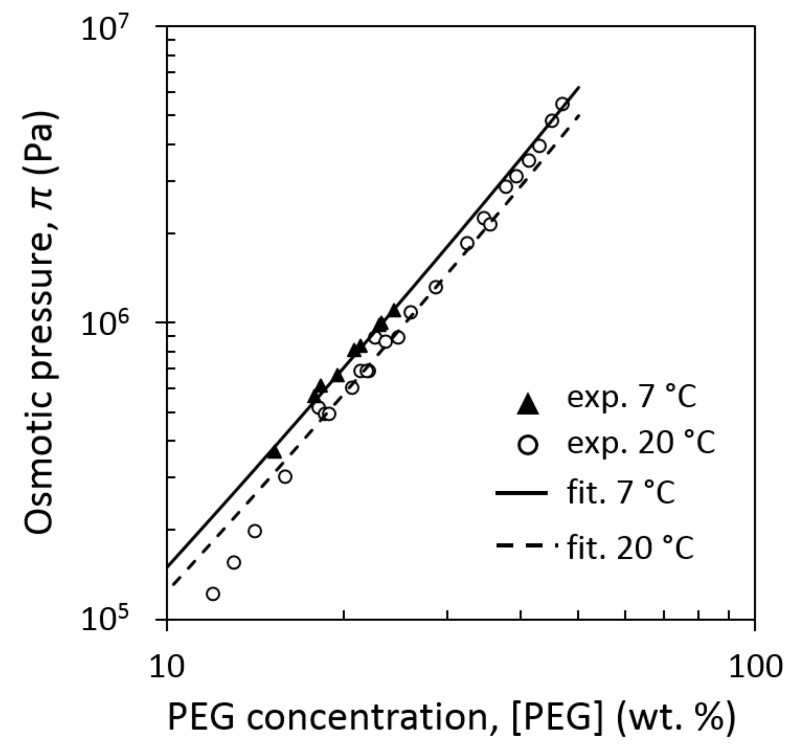
Osmotic pressure versus concentration of polyethylene glycol (PEG) 20 kDa. Experimental results of Rand website [41] (symbols) and least square fits with the help of Equation (2) (curves).

In the literature, there is no equation relating *π* and *C* for PEG at 7 °C. Only experimental data provided on a website by [41] (data are reported verbatim from the website in the supplementary material S.1.) are available for PEG 20 kDa at 20 and 7 °C. These data were fitted with the help of Equation (2), and are presented in Figure 1 together for the same PEG at 20 °C. For the data obtained at 7 °C, the value of temperature-dependent parameter $\bar{V}$ = 0.813 mL/g, which is required for the fitting, was derived from the experimental $\bar{V}\left(T\right)$ data of a previous work [42] by extrapolation, and the value of *α* was obtained by the fitting.

Application of Equation (2) results in a fairly good fitting of experimental data on Rand website [41] both for 7 °C and for 20 °C. For 7 °C, the obtained value of fitting parameter is *α* = 0.573. It was assumed that at 7 °C the value of *α* determined by the fitting for 20 kDa is equal to that for 35 kDa PEG as soon as *α* only slightly changes in this range of molecular weight, as predicted from Equation (3). Therefore, the value *α* = 0.573 was used for the calculation of osmotic pressure with the help of Equation (2) at 7 °C for both 20 and 35 kDa PEG solutions in our experiments.

Previous works [40,43] compared osmotic pressures of silica dispersions obtained by osmotic stress technique with PEG 35 kDa with those theoretically calculated via Monte Carlo simulations. They showed a discrepancy of a factor of around two between the results of simulation and the experimental data when the latter was treated with the help of Equation (1). They also showed that there was a perfect match of experimental data with the theoretical predictions if Equation (2) was used for the data treatment. They explained this difference by a flaw in the experimental data of Bouchoux et al. [14] that resulted in erroneous values of fitting parameters of Equation (1) for the stressing polymer PEG 35 kDa. This error does not influence general conclusions of previous studies [13,14,15,16,26]. However, in the present study, Equation (2) was used in order to determine osmotic pressures from PEG equilibrium concentrations.

#### 2.2.3. Experimental Protocol

Standard regenerated cellulose Spectra/Por 2 dialysis bags of 16 and 29 mm diameters with a cut-off of 12–14 kDa were used (Repligen, Waltham, Mass, USA), which would mimic ultrafiltration. These bags and their cut-off were chosen so that they would only retain the polymer and the colloidal matter of the sample (casein micelle). The small species such as lactose (342 Da) and ions are free to pass through the membrane and equilibrate in both sides of the membrane. Dialysis bags were washed with UF permeate and conditioned during at least thirty minutes in UF permeate. Casein micelle liquid dispersion at 20 g/L was used to reach low concentrations from 20 to 100 g/L. Casein micelle liquid dispersion at 100 g/L was used to reach high concentrations from 100 to 700 g/L. Those casein micelle dispersions were introduced into thoroughly closed dialysis bags and then immersed in the stressing solutions kept at 7 °C or 20 °C under a low agitation of 100 rpm. In order to obtain a sufficient amount of concentrated dispersion, the bags were refilled with casein micelle dispersions several times during 2 days for low concentrations and 7 days for high concentrations. The polymer stressing solution was changed after 7–15 days for high concentrations to limit its dilution and/or pH variation. The target pH was 6.7 ± 0.1.

To avoid the proteolysis of casein micelles that occurs after 7 days of dialysis for osmotic stress under 5 kPa [14], for low concentrations, osmotic stress experiments performed at low pressures were equilibrated during 7 days. A measurement after 14 days, similar to the one obtained after 7 days, confirmed that the equilibrium was effectively reached after 7 days. For osmotic stress beyond 5 kPa, for high concentrations, since the concentration was quicker, the proteolysis was negligible [14], so the samples were equilibrated during 30 days.

All measurements were repeated at least two times.

#### 2.2.4. Casein Concentration and Effective Volume Fraction Calculations

After equilibrium was reached, the casein concentration in dialysis bags [Cas] (in wt. %) was calculated with the help of Equation (4) (see Supplementary material S.2. for complete definition):

(4) $$\left[\mathrm{Cas}\right]=\frac{TS_{powder}\cdot \left(1-NCN\right)\cdot \left(TS_{disp}-TS_{uf}\right)}{\left(1-TS_{uf}\right)}$$

where *TS_powder_, TS_disp_*, and *TS_uf_* are the total solid contents in the powder, in the dispersion, and in the UF permeate, in wt. %, respectively, and *NCN* is the amount of non-casein nitrogen matter in the isolate casein powder (Table 1). The total solid contents in the casein isolate powder, all dispersions, and UF permeate were obtained by weighing the sample after drying for 7 h at 105 °C [44].

The casein concentration in the sample (expressed in g of casein per L of sample) is then calculated as

(5) $${\left[\mathrm{Cas}\right]}_{g/L}=\frac{1000}{0.733+\left(1-TS_{disp}\right)/\left[\mathrm{Cas}\right]}$$

For the derivation of Equation (5), it is assumed that dispersion consisted in a dispersion medium, having density of pure water, and casein, which has a density that is inverse to its weighted average partial specific volume, 0.733 mL/g. This value is determined from the individual casein amino acid sequences [45].

Finally, the effective volume fraction *φ_eff_* of casein micelles in dispersion is defined as:

(6) $$\phi_{eff}=v{\left[\mathrm{Cas}\right]}_{g/L}$$

where *v* is the apparent voluminosity of casein micelle equal to 4.85 mL/g at 7 °C and 4.1 mL/g at 20 °C [20] for pasteurized skim milk. It should be noted that other authors took a different value of apparent voluminosity, such as *v* = 4.4 mL/g at 20 °C, which is the value for native (nonpasteurized) casein micelles [5,14,16]. As the casein isolate powder used in the current study was obtained from pasteurized milk, the values of apparent voluminosity reported by [20] were used in the following calculations.

The so-called effective volume fraction gives an indication of the deformation and compression experienced by casein micelles at high concentrations [14]. When *φ_eff_* > *φ_eff_*_,*max*_, where *φ_eff,max_* is the volume fraction at random close-packing of casein micelles, *φ_eff,max_* = 0.68–0.8 [14,16,17,18,19], and casein micelles start to be partially deformed. Then, *φ_eff_* > 1 indicates that the casein micelles are compressed to a lower volume, that is, partially de-swelled and compressed.

### 2.3. Rheology

Rheological behavior of concentrated casein micelle dispersions was studied through flow and oscillatory experiments. Samples were either prepared by dissolution of casein isolate powder in UF permeate for concentrations below 140 g/L or prepared by osmotic stress in dialysis bags of 45 mm diameter for concentrations above 140 g/L. Steady shear viscosities were measured for liquid-like casein micelle dispersions, say in the compression regimes 1 and 2. Oscillatory experiments were performed for dispersions in a large range of concentrations from 101 g/L to 350 g/L, being in the regimes 2 and 3.

Flow measurements were performed using a Low Shear 400 viscometer (Lamy Rheology, Champagne au Mont d’Or, France) using a Couette geometry (inner and outer radii = 5.5 and 6.0 mm, shear rate = 0.1208–120 s^−1^) for dispersions from 101 to 135 g/L and a DHR2 rheometer (TA Instruments France, Guyancourt, France) with a cone–plate geometry (diameter = 25 mm, angle of 2°, shear rate = 0.1 to 1000 s^−1^) for dispersions from 147 to 174 g/L. The apparent viscosity of the UF permeate, defined as $\eta_{s}$, was measured with the Low Shear 400 and was 1.795 mPa∙s at 7 °C and 1.242 mPa∙s at 20 °C.

The rheometric measurements were performed at the same temperature as for production of casein micelle dispersions, either at 20 or at 7 °C. A closed cover and mineral oil were used in order to prevent evaporation.

Apparent viscosity was measured at increasing shear rates at steady state, that is, after a time of stabilization between 1 and 20 min. On the DHR2 device, after the measurement at the highest shear rate, measurements at 3 and then 30 s^−1^ were performed to check if the initial sample structure was irreversibly damaged by the measurements. It was concluded that the samples were not damaged irreversibly since values obtained after the shear rate decreasing to 3 and 30 s^−1^ were consistent with that obtained during shear rate increasing.

The dimensionless relative apparent viscosity of dispersions was calculated as

(7) $$\eta_{r}=\;\eta /\eta_{s}$$

where *η* is the sample apparent viscosity (Pa∙s).

Oscillatory shear measurements were performed with the DHR2 rheometer using the same cone–plate geometry as above. At higher concentrations, the dispersions behaved as solids, and parallel plates with grooved surfaces (diameter = 20 mm) were used. Solid-like samples were cut in cylindrical pieces of 2 cm of diameter and 4 mm height at the studied temperature. Then, the sample was quickly transferred onto the lower plate, and the upper plate was gently lowered until the sample filled the gap and a constant normal force of around 0.2 N was reached. The sample initially stored at the measured temperature (7 °C or 20 °C) was conditioned at rest at the corresponding studied temperature during 10–15 min before measurement. Viscoelastic moduli were first measured as a function of stress at the frequency of 1Hz. A strain of 1%, located in the linear viscoelastic region, was applied in a frequency sweep (10–0.1 Hz).

All measurements were repeated at least two times.

## 3. Results

### 3.1. Equation of State of Casein Micelle Dispersions

#### 3.1.1. Equation of State at 20 °C

**Figure 2:**
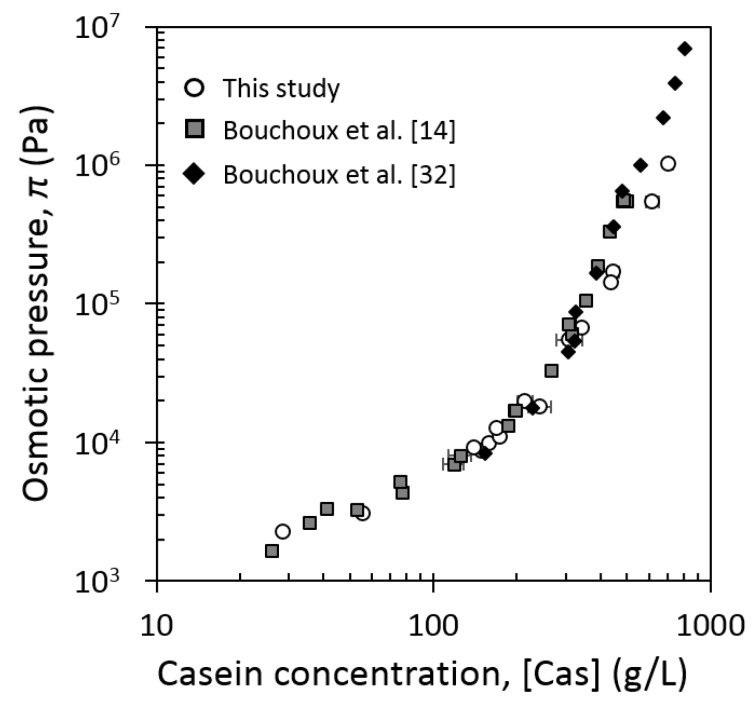
Osmotic pressure of casein micelle dispersions in UF (ultrafiltration) permeate as a function of casein concentration at 20 °C. Results of the current work with casein isolate powder obtained from pasteurized milk (open circles); results of [14] recalculated with Equation (2) for native (nonpasteurized) casein isolate (called Native PhosphoCaseinate, NPC) (solid squares); results of [26] recalculated with Equation (2) for nonpasteurized NPC (solid diamonds).

In order to compare in details our data at 20 °C with those from previous publications, osmotic pressures of Bouchoux et al. [14,26] have been recalculated by using Equation (2) and are represented in Figure 2. The π([Cas]) dependency (Figure 2) followed the typical trend with three compression regimes already described in literature [14]. In the current work,

(1) until around 170 g/L, osmotic pressure was proportional to the casein concentration, $\pi \propto {\left[\mathrm{Cas}\right]}_{g/L}^{1}$, which corresponds to the dilute regime, where casein micelle dispersions are liquids;
(2) from 170 to 214 g/L, osmotic pressure was no longer directly proportional to casein concentration and started to rise faster, which corresponds to the transition phase, where casein micelle interactions become stronger;
(3) beyond 214 g/L, osmotic pressure rose much faster, as around the sixth power of casein concentration, $\pi \propto {\left[\mathrm{Cas}\right]}_{g/L}^{6}$, that corresponds to the concentrated regime, where casein micelles behave as soft solids.

Our results were similar to those from the literature [14,26] for casein concentrations from 25 to around 350 g/L. But beyond 350 g/L, that is, in the concentrated regime, casein micelle dispersions studied in the current work became more concentrated (by a factor of about 1.3) than those studied previously at the same values of osmotic pressure. It means that casein micelles used in the current work were easier to concentrate than those studied by previous authors [14,26].

#### 3.1.2. The influence of temperature on osmotic pressure of casein micelle dispersions

**Figure 3:**
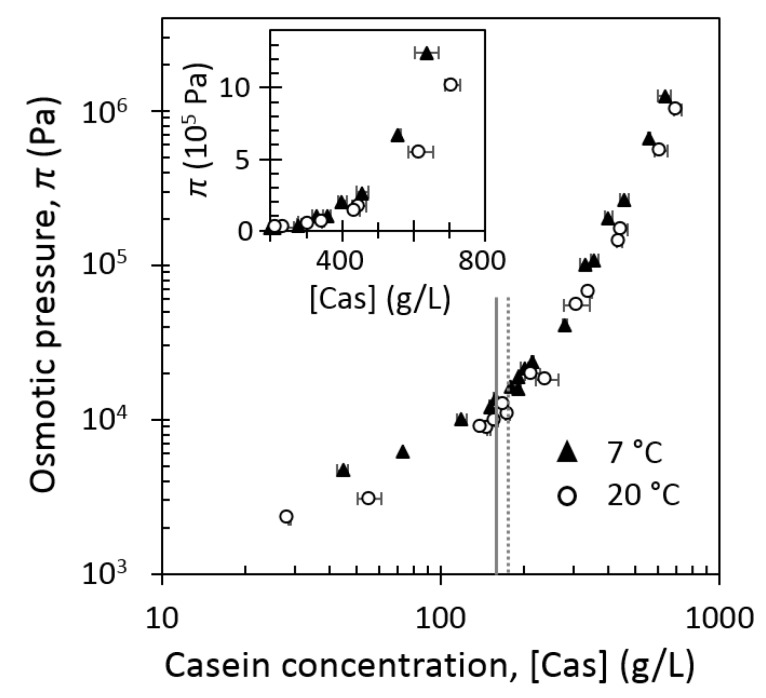
Osmotic pressure, *π*, of casein micelle dispersions in UF permeate at 7 °C (solid triangles) and at 20 °C (open circles) as a function of casein concentration (in g/L), vertical lines correspond roughly to changes of slope at 7 °C (solid line) and 20 °C (dashed line). Insert details high-concentration regions in linear coordinates.

Figure 3 shows the comparison of the equations of state of casein micelles at 7 °C and 20 °C.

As well as at 20 °C (Figure 2), three different compression regimes could be identified at 7 °C (Figure 3). However, at 7 °C, the corresponding border concentrations, that is, at slope changes, seemed to be shifted to lower concentrations, as compared with those at 20 °C. At 7 °C, the border between the dilute and the transition regimes was located around 150 g/L, while concentrated regime began at around 170 g/L.

Regardless of the π value, casein concentration was higher at 20 °C than at 7 °C. This difference increased at high casein concentration (insert in Figure 3). At 7 °C, casein micelle dispersions needed an osmotic pressure twice higher than at 20 °C in order to reach the concentration of 600 g/L. This emphasized novel important information: at 20 °C casein micelles were easier to concentrate than at 7 °C.

### 3.2. Influence of Temperature on Rheology and Sol–gel Transition of Casein Micelle Dispersions

#### 3.2.1. Flow Measurements

##### Influence of Temperature on Viscous Flow

**Figure 4:**
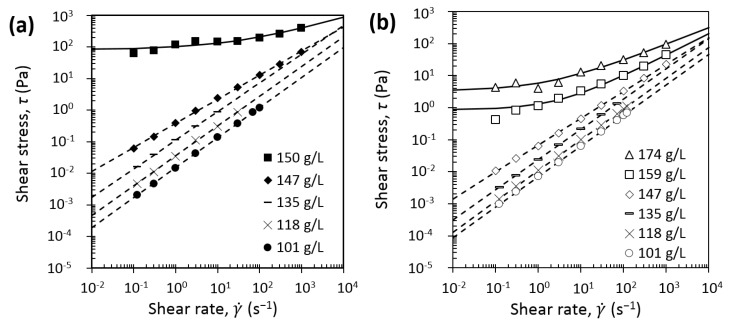
Flow curves of casein micelle dispersions in UF permeate at (**a**) 7 °C and (**b**) 20 °C. Dashed lines correspond to power law fitting with Equation (8), and solid lines correspond to Herschel–Bulkley law fitting with Equation (9). The rheometric measurements were performed at the same temperature as the one for fabrication of casein micelle dispersions, respectively at 7 °C and at 20 °C.

The influence of temperature on the flow properties of casein micelles dispersions has been investigated by rheometric measurements (Figure 4) performed at the same temperature as the one for production of dense dispersions, respectively at 7 °C (Figure 4a) and 20 °C (Figure 4b).

The steady state flow curves (Figure 4) exhibited changes in rheological behaviors with the increase of casein micelles concentration. A change from Newtonian to shear thinning, and then to a yield stress fluid is evidenced at increasing casein micelle concentrations regardless of the measured temperature (7 °C and 20 °C), which is in a good agreement with the literature data at 20 °C [16,17,18,19]. Particularly, changes in rheological behaviors from shear thinning to a yield stress behavior is evidenced by a great difference between 147 g/L flow curve and 150 g/L flow curve at 7 °C and 159 g/L flow curve at 20 °C due to the appearance of a viscoelastic solid behavior. For the two studied temperatures, at the lowest studied casein micelle concentration, [Cas] = 101 g/L, the dispersions behaved quite as Newtonian fluids over the studied range of shear rates with slopes of 0.95 at 7 °C and 0.96 at 20 °C. At higher concentrations (118, 135, and 147 g/L), casein micelle dispersions at both 7 and 20 °C exhibited a shear thinning behavior. At higher concentrations, a yield stress was detected from 150 g/L for the dispersions measured at 7 °C (Figure 4a) and from 159 g/l for the dispersions measured at 20 °C (Figure 4b). Remarkably the yield stress measured at 20 °C at 159 g/L was equal to 1 Pa, which is significantly lower than the one measured at 7 °C, equal to 83.2 Pa, even at a lower concentration of 150 g/L. This huge difference in the two yield stress values measured at the two equilibrium temperatures would certainly be linked to a deep change in the structural organization or internal mutual interactions within the casein micelle dispersions. In order to better quantify these changes, flow curves (Figure 4) were fitted with rheological models such as the power (Equation (8)) or the Herschel–Buckley (Equation (9)) law, depending on the casein micelle concentration and conditioning temperature. Shear thinning at high shear rates is a common phenomenon and has already been pointed out by previous works for casein dispersions at 20 °C [16,18]. This behavior can be described by a power law:

(8) $$\tau \;=\;K\;{\dot{\gamma }}^{n}$$

where τ is the shear stress (Pa), *K* is the consistency (Pa∙s^n^), $\dot{\gamma }$ is the shear rate (s^−1^), and *n* is the shear-thinning index. This model was successfully applied to our experimental data (dashed lines in Figure 4). At higher concentrations, dispersions exhibited a gel consistency and yield stresses emerged on the flow curves. In this case, the Herschel–Bulkley viscoplastic model was used for curve fitting:

(9) $$\tau \;=\tau_{0}+\;K\;{\dot{\gamma }}^{n}$$

where $\tau_{0}$ is the yield stress (Pa). The results of the fitting are presented in Figure 4 by solid curves.

##### Influence of Temperature on Apparent Viscosity of the Solvent

**Figure 5:**
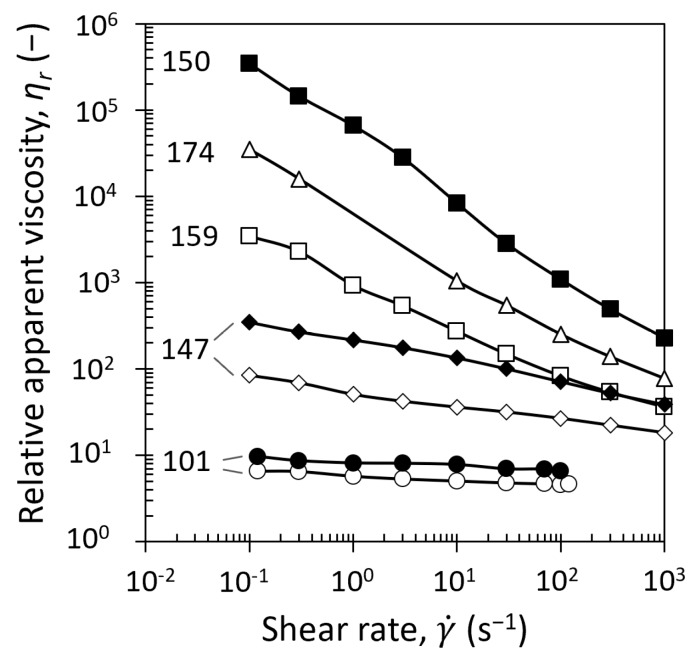
Shear rate dependence of relative apparent viscosity for casein micelle dispersions in UF permeate at 7 °C (solid symbols) and at 20 °C (open symbols). Casein dispersions concentrations [Cas]_g/L_ are shown near the curves. The curves serve as a guide to the eye. The rheometric measurements were performed at the same temperature as the one for fabrication of casein micelle dispersions, respectively at 7 °C and at 20 °C.

Another way to present the effect of temperature on the rheological properties of casein micelles dispersions is to calculate from the rheometric data of Figure 4 the relative apparent viscosity as a function of the shear rate applied (Figure 5).

Application of relative apparent viscosity allows to eliminate the explicit influence of the temperature on the apparent viscosity of the solvent phase. For the studied range of casein concentration, relative apparent viscosities of casein micelle dispersions were higher at 7 °C than at 20 °C, which reinforced the conclusions obtained on the changes in yield stress values for these dispersions.

##### Changes in Consistency and Shear-Thinning Index at 7 °C and 20 °C

**Figure 6:**
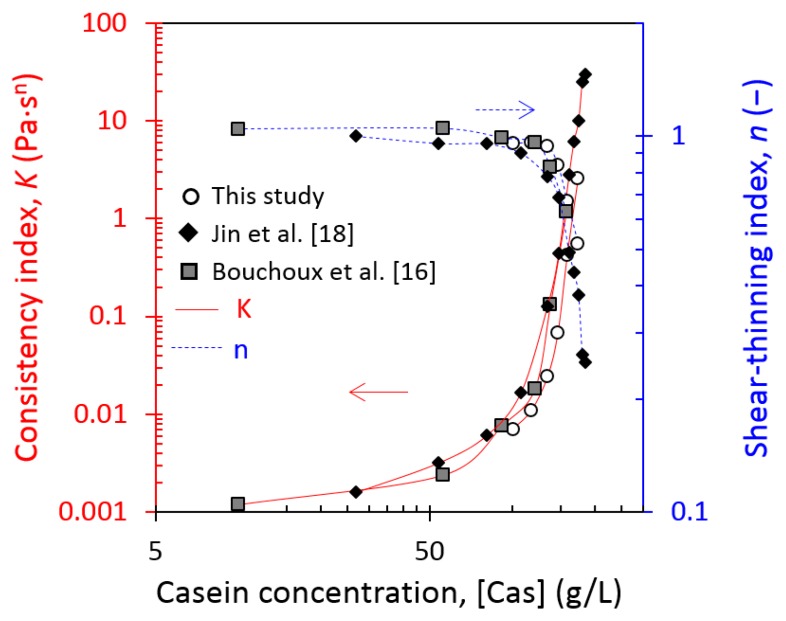
Consistency, *K* (solid red curves), and shear thinning index, *n* (dashed blue curves), as a function of casein concentration at 20 ± 3 °C obtained in this work (open circles), by [16] (solid squares) and [18] (solid diamonds). The curves serve as guides to the eye. (For interpretation of the references to color in this figure, the reader is referred to the web version of this article).

Figure 6 represents the fitted parameters of rheological models as a function of the casein concentration obtained at 20 °C in the current work in comparison with previously published data [16,18].

Regardless of the authors, results were quite similar. The values of consistency and shear-thinning index remained respectively low and stable (K under 1 and n close to 1) until around 160 g/L. Beyond this critical concentration, the consistency increased and the shear-thinning index decreased strongly with the increase in the casein concentration. Jin et al. [18] determined an estimated value of sol–gel transition concentration of 185 g/L through a percolation law on their yield stress data. Unfortunately, Bouchoux et al. [16] and the current work did not present sufficient experimental points beyond 170 g/L to determine the sol–gel transition concentration through a percolation law. Another method used by Bouchoux et al. [16] has been preferred to determine sol–gel transition concentration and will be presented in Section 3.2.2 (Determination of Sol–Gel Transition). Regardless of the methods used, a same sol–gel transition at 178–185 g/L was obtained.

Despite the use of different casein micelle materials, the trend of the current work at 20 °C was quite similar to that in the literature. In these experiments, Bouchoux et al. [16] used “native” casein isolate powder (manufactured from nonpasteurized milk and called Native PhosphoCaseinate, NPC by the authors) solubilized in a UF permeate, and Jin et al. [18] used the same casein isolate powder as in the current work, but solubilized in water.

**Figure 7:**
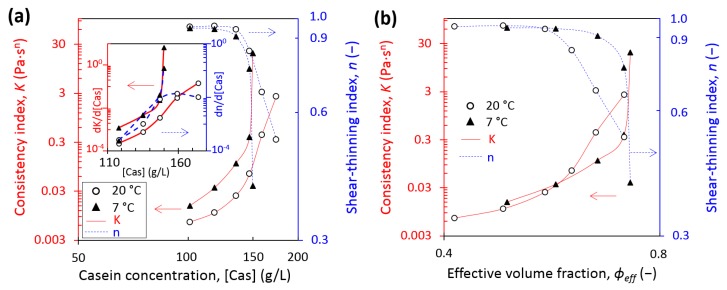
Consistency, *K* (solid red curves), and shear thinning index, *n* (dashed blue curves), at equilibrium conditioning temperature of 7 °C (solid triangles) and at 20 °C (open circles) as a function of (**a**) casein concentration and (**b**) casein effective volume fraction. The curves serve as guides to the eye. Insert details derivatives of K and n within the concentration. (For interpretation of the references to color in this figure, the reader is referred to the web version of this article).

Figure 7a shows the comparison of consistency and shear-thinning index at equilibrium conditioning temperatures of 7 and 20 °C as a function of the casein concentration. At 7 °C, 150 g/L appeared as the critical concentration of the strong increase of K and the decrease of n and was lower than at 20 °C, where it was around 160 g/L. The respective decrease of shear thinning index and increase of consistency index were quite soft and stable at 20 °C at the critical value compared with at 7 °C, where they were very strong, as detailed in the insert by the derivatives of K and n on casein concentration. Figure 7b takes into account the correction of casein micelle apparent voluminosity by plotting the consistency and the shear-thinning index versus the casein volume fraction at 7 °C and 20 °C. Therefore, from our results at 7 °C and 20 °C, an effective volume fraction of around 0.7–0.72 corresponded to the critical value of a behavior change. This estimated value is in accordance with the literature in the range of 0.68–0.8 [16,17,18,19,20]. However, depending on the authors, the voluminosity used for the calculation of the effective volume fraction is different. By using sol–gel transition concentration determined by Bouchoux et al. [16] and Jin et al. [18], respectively 178 g/L and 185 g/L, and using a voluminosity of 4.1 mL/g at 20 °C, the effective volume fraction was equal, respectively, to 0.73 and 0.76, values very close to the value found in this work.

#### 3.2.2. Oscillation Measurements

##### Viscoelastic Properties in the Sol–gel Transition and Above

**Figure 8:**
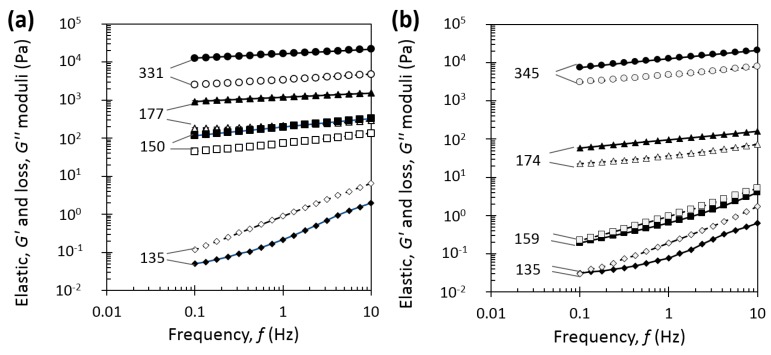
Frequency dependence of the elastic G’ (solid symbols) and loss G” (open symbols) moduli for casein micelle dispersions in UF permeate at different casein concentrations at equilibrium conditioning temperature of (**a**) 7 °C and at (**b**) 20 °C. Casein dispersions concentrations [Cas]_g/L_ are shown near the curves.

Figure 8 shows the frequency dependence of the elastic modulus G’ and the loss modulus G” of casein micelle dispersions with casein concentrations ranging from 137 to 338 g/L. For better readability, only a few concentrations are presented. At [Cas] = 135 g/L, casein micelle dispersions behaved as viscoelastic liquids at 7 °C and 20 °C with a strong frequency dependence. Then, at 7 °C (Figure 8a), at the concentration of 150 g/L, results showed a dominance of the elastic modulus and a much lower frequency dependence than at 135 g/L. At 20 °C (Figure 8b), the change of regime with the dominance of elastic modulus did not happen yet at 159 g/L, but at least at 174 g/L. At the highest concentrations, the dispersions at 331 g/L at 7 °C and 345 g/L at 20 °C behaved as viscoelastic solids and exhibited typical weak gel-type mechanical spectra, with G’ clearly dominating G” in the entire studied frequency range, and moduli slightly varied on frequency [16]. Indeed, at these highest concentrations at 7 °C and 20 °C, $G^{\prime }\propto f^{0.1}$ and $G^{\prime }\propto f^{0.2}$, respectively, in comparison with solutions at low concentrations, namely 136 g/L that exhibits $G^{\prime }\propto f^{2}$ [16]. Anyway, at very low frequencies, say at 3.10^−5^ Hz, a cross-over between G’ and G’’ for dispersions at 370 g/L has been shown with creep/recovery experiments, which demonstrates that these physical gels do not behave as true elastic gels [16]. Therefore, at 7 °C, the change of regime from viscoelastic liquid to viscoelastic solid happened at a lower concentration than at 20 °C.

##### Determination of Sol–Gel Transition

**Figure 9:**
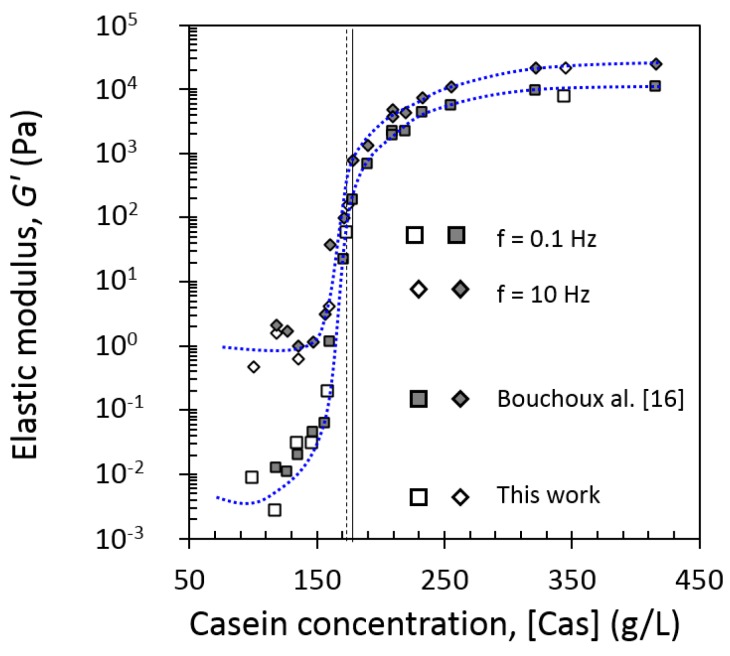
The low-frequency (f = 0.1 Hz, squares) and high-frequency (f = 10 Hz, diamonds) elastic moduli of casein micelle dispersions in UF permeate as a function of casein concentration at 20 °C. Results of this work (open symbols) are compared with those of the literature (solid symbols) [16]. Dashed line corresponds to sol–gel transition concentration at [Cas] = 174 g/L. Solid line corresponds to sol–gel transition concentration at [Cas] = 178 g/L. The dotted curves serve as guides to the eye.

From Figure 8, elastic and loss moduli at low frequency (0.1 Hz) and high frequency (10 Hz) were extracted for each concentration. Figure 9 gives the concentration dependence at 0.1 Hz and 10 Hz of the elastic modulus at 20 °C from the current work and from literature [16]. Loss modulus followed the same trend as elastic modulus (results not shown). Curves at both frequencies were very similar and showed a strong increase of elastic modulus with the increase of casein concentration in the range ~145 to ~180 g/L. At [Cas] = 174 g/L in the current work, the elastic G’ moduli at 0.1 and 10 Hz have come closer compared with at the low concentrations, and from this point, they remained in the same ratio. This change in frequency dependence at [Cas] = 174 g/L in the current work was taken as the “gel point” of the dispersion, and it was in great agreement with previous work finding the “gel point” by this method at [Cas] = 178 g/L [16]. It was also similar to the sol–gel transition concentration found by other authors equal to 185 g/L [18].

**Figure 10:**
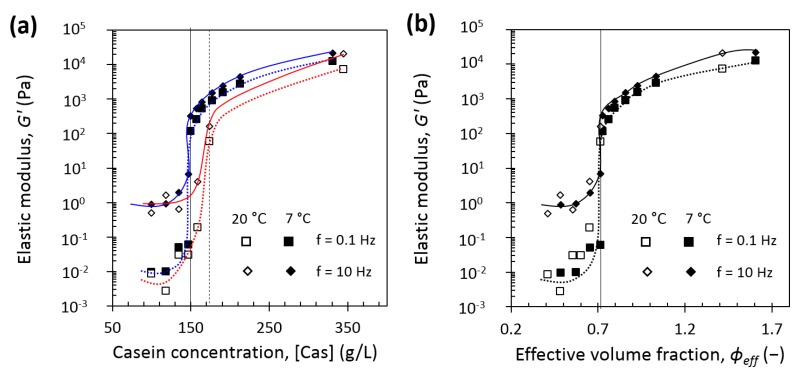
The low-frequency (f = 0.1 Hz, squares) and high-frequency (f = 10 Hz, diamonds) elastic G’ modulus of casein micelle dispersions in UF permeate at equilibrium conditioning temperature of 7 °C (solid symbols) and at 20 °C (open symbols) as (**a**) a function of casein concentration. Vertical lines correspond to sol–gel transition concentration of [Cas] = 150 g/L at 7 °C (solid line) and [Cas] = 174 g/L at 20 °C (dashed line); (**b**) a function of effective volume fraction. Solid line corresponds to *φ_eff max_* = 0.71. The solid and dotted curves serve as guides to the eye.

Figure 10a gives the concentration dependence of the elastic modulus at 0.1 and 10 Hz for casein micelle dispersions at equilibrium conditioning temperatures of 7 °C and 20 °C. In both cases, G’ at 0.1 and 10 Hz strongly increased when casein concentration increased. But the steep increase of the modulus happened at a lower range of concentration from 135 to 150 g/L at 7 °C compared with 145–174 g/L at 20 °C. In the same way as above, elastic moduli at 0.1 and 10 Hz have come much closer at a lower concentration equals to around 150 g/L at 7 °C against around 174 g/L at 20 °C and beyond this concentration, they remained in the same ratio. We took those ranges of concentrations as “gel points” at 7 and 20 °C, and they were confirmed by plotting slopes of elastic modulus over frequency as a function of casein concentration (see Supplementary material S.3.). Figure 10b takes into account the correction of casein micelle apparent voluminosity at 7 and 20 °C and gives the casein effective volume fraction dependence of the elastic modulus obtained at 0.1 and 10 Hz for casein micelle dispersions at 7 °C and 20 °C. Such a correction revealed that at 7 and 20 °C, the steep increase of the moduli is observed at the same particle volume fraction of *φ_eff max_* = 0.71, which is in great agreement with the previously reported data for ambient temperature of 0.68–0.8 [14,16,17,18,19,20,46] or even of the recalculated effective volume fraction of 0.73 and 0.76 for the respective works of Bouchoux et al. [16] and Jin et al. [18] with a voluminosity of 4.1 mL/g at 20 °C.

## 4. Discussion

### 4.1. Influence of the Nature of Casein Micelles Dispersions on Their Compressive and Rheological Properties at 20 °C

Previous works [14,16,18] studied compressive and rheological properties of casein micelle dispersions at 20 °C. Bouchoux et al. [14,16] used NPC powder (“native” casein isolate powder manufactured from nonpasteurized milk) solubilized in a UF permeate, and Jin et al. [18] used the same casein isolate powder as in the current work (casein isolate powder manufactured from pasteurized milk), but solubilized in water. The current work consolidated the results previously obtained by these authors but highlighted also some differences in the concentrated regime.

The results obtained in the current work showed that, below a casein concentration of 350 g/L and regardless of the nature of the casein micelle dispersions used in the experiments, the compressive and rheological properties of the casein micelles did not show great differences: the equation of state of casein micelle dispersions of the current work at 20 °C was identical to the one of NPC dispersion under 350 g/L (Figure 2); the values of consistency and shear-thinning indexes (casein concentration <200 g/L, Figure 6), the sol–gel transition and elastic G’ moduli (casein concentration <350 g/L) were a bit different but in the same order of magnitude and were quite consistent with values from these previous works.

On the contrary, beyond 350 g/L, at 20 °C (Figure 2), casein micelle dispersions used in the current work were more easily concentrated than native casein micelles (NPC) used by Bouchoux et al. [14]. The NPC powder contains a lower content of small species, such as serum proteins (4.0–5.1% Total solids (TS)), compared with the casein powder used in the current work (7.2% TS), but no difference in casein dispersions were observed in the dilute regime from 25 to 150 g/L (Figure 2), where the osmotic pressure of the dispersion is dominated by the contribution from small peptides [14]. As a result, the potential higher content of serum proteins in our dispersions had no significant effect on osmotic pressure changes, and the difference in compositions of small species (serum proteins, lactose, etc.) contained in the casein powders cannot explain the observed differences in compressive properties. The cause of difference of compressibility could likely be attributed to difference in casein micelles structure, resulting from the difference of casein micelle preparation and heat treatment (pasteurization in our case) applied to the milk prior to microfiltration. High pasteurization temperature results in a denaturation of whey proteins that partly associate with casein micelles [47,48], but such an association does not seem conceivable in our work since the pasteurization is probably done at low time and temperature, where too few whey proteins associate with casein micelles [47,48]. Some studies suggest that increasing storage temperature implies a decrease of hydration and voluminosity of casein micelles [20,24], but a change of total voluminosity of casein micelles is an unlikely possibility because such change in the structure of casein micelles would have been observed on the whole concentrated regime and in the transition regime of the equation of state, and not only in the very high concentrations (i.e., where casein micelles are deformed and de-swelled). However, heat treatment of milk is also responsible for the decrease of soluble calcium phosphate, CaP, and therefore for the increase in CaP nanoclusters within the micelles [49]. Casein micelles are constituted of dense regions made of nanoclusters of CaP and void regions that are filled with water [2,13]. The higher the number of CaP nanoclusters in the casein micelle, the higher the number of dense regions, resulting in less water in the casein micelle core and, therefore, this could lead to a higher ability to lose water when casein micelles are compressed at high pressures.

Therefore, differences of compressibility at high concentrations are likely due to small differences of structure and composition induced by pasteurization, especially water content of casein micelle core that is revealed during compression. But investigations to support this conclusion are still needed.

### 4.2. Influence of the Equilibrium Conditioning Temperature of Casein Micelle Dispersions on Their Compressive and Rheological Properties

An interesting result is the fact that the equilibrium conditioning temperature (7 °C or 20 °C) impacted the compressibility and rheological behavior of casein micelle dispersions. As a reminder, the structure of casein micelles undergoes modifications induced by a low temperature (7 °C): hydrophobic interactions are weakened, β-casein and nanoclusters of CaP are released, and thus more water can enter into casein micelles, implying a rise in their hydration, diameter, and voluminosity.

The differences observed at 7 °C and 20 °C will be examined in the three concentration regimes defined previously in Section 3.1: dilute, transition, and concentrated regimes.

At low casein concentration (dilute regime), casein micelle dispersions behaved as Newtonian fluids at both 7 °C and 20 °C and the apparent viscosity of casein micelles dispersions was higher at 7 °C than at 20°C. Equilibrium conditioning at 7 °C led also to dispersions that were less easy to concentrate compared with at 20 °C (Figure 3).

**Figure 11:**
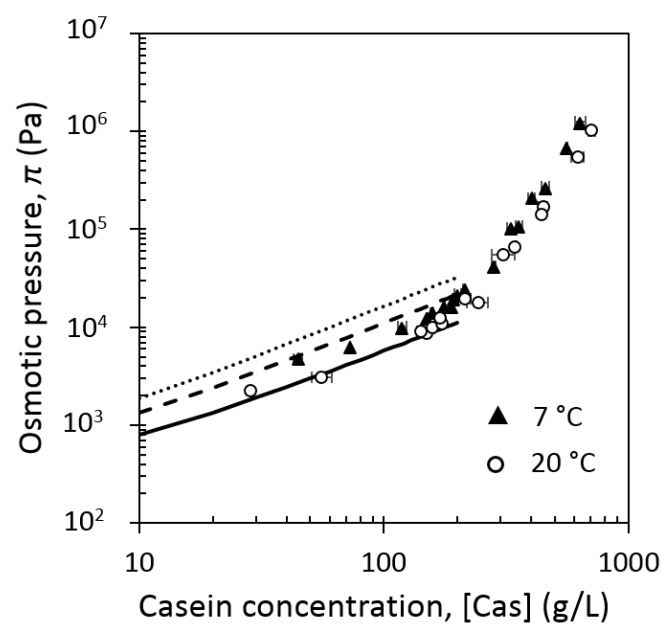
Osmotic pressure of casein micelle dispersions in UF permeate as a function of casein concentration at 7 °C (solid triangles) and at 20 °C (open circles and solid line) and the predictions of van’t Hoff law in the dilute regime. The osmotic pressures were calculated through Equation (10), using the estimated number concentrations of experimental osmotic pressure at 20 °C and β-casein release estimated to 20% of total β-casein (dashed line) and experimental osmotic pressure at 20 °C and β-casein release estimated to 40% of total β-casein (dotted line).

In this range of concentration, the osmotic pressure only measures the numbers of species and is the sum of contributions from all the noninteracting species in the dispersion (van’t Hoff law). The shift of the equation of state observed in this regime between 20 °C and 7 °C could be attributed to the release of free soluble casein, mainly β-casein at 7 °C. Indeed, a release of around 35% of total β-casein after 50 h at 4 °C has been reported [9]. Similarly to Bouchoux et al. [14], the contribution of β-casein on the osmotic pressure can be estimated by using the Van’t Hoff law:

(10) $$\pi =RT\;\underset{i}{\sum }C_{i}$$

with $C_{i}$ the concentrations of non-interacting species i, expressed in moles per unit, R, the universal gas constant (J∙mol^−1^∙K^−1^) and T, the temperature (K). We have estimated the osmotic pressure by using two simulations of β-casein release (Figure 11): (i) a simulation with a “low” release of 20% of β-casein; (ii) a simulation with a “high” release of 40% of β-casein to overestimate it. For the simulations, β-casein at 7 °C is considered as a molecule from 7 to 8 nm [50]. “Low” or “high” contribution (20% and 40%) of β-casein was added to the contribution of experimental osmotic pressure at 20 °C extrapolated to the solid line. Figure 11 shows that osmotic pressure calculated with the “low” contribution reached experimental osmotic pressure at 7 °C, whereas the “high” contribution overestimated experimental osmotic pressure at 7 °C. Therefore, the release of β-casein in the solvent phase could explain the shift between the two equations of state at 7 °C and 20 °C in the dilute regime.

Moreover, in the dilute regime, the differences of apparent viscosity observed between 7 °C and 20 °C could not be attributed to the difference in solvent apparent viscosity with the temperature change, as shown in Figure 5. The differences in relative apparent viscosities could be explained more probably by an increase of voluminosity of the casein micelles induced by a decrease of temperature, as a temperature of 7 °C results in higher hydration and higher voluminosity of the casein micelle. The relative apparent viscosity in function of casein concentration or volume fraction is usually modeled with Quemada’s equation:

(11) $$\eta_{r}={\left(1-\frac{\phi_{eff\;}}{\phi_{eff\;max}}\right)}^{-2}$$

**Figure 12:**
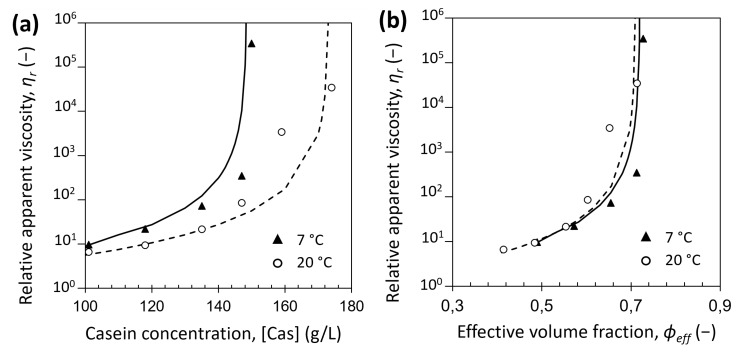
Relative apparent viscosity at low shear (0.1 s^−1^) of casein micelle dispersions in UF permeate at 7 °C (solid triangles) and at 20 °C (open circles) as a function of (**a**) casein concentration and (**b**) effective volume fraction. The solid and dashed lines are calculated through the Quemada’s equation, Equation (11), with ϕ_eff max_ = 0.71 at 7 °C and 20 °C, respectively.

In our case, with ϕ_eff max_ = 0.71, η_r_ at 7 °C and 20 °C follows Equation (11), respectively with variations of around 30% and 20%–50% in comparison with experimental data (Figure 12a). Figure 12b shows that in the dilute regime, when casein micelle apparent voluminosity is taken into account, a unique Quemada’s fit is obtained at 7 °C and 20 °C. Therefore, a decrease of temperature implies that casein micelles take more space at 7 °C than at 20 °C, resulting in an increase of relative apparent viscosity at the same casein concentration.

In the transition regime, casein micelles start to be in contact with each other and a sol–gel transition is observed, due to attractive attraction overcoming repulsive interactions between κ-casein brushes [14]. In this regime, regardless of the temperature, casein micelle dispersions exhibited a shear-thinning behavior and a yield stress in the transition regime. But remarkably, changes in shear-thinning and consistency behavior (Figure 4 and Figure 7a), yield stress values (Figure 4), and shift from viscoelastic liquid to viscoelastic solid (Figure 8) were observed at lower concentrations and with stronger intensities at 7 °C compared with 20 °C. Both the osmotic stress experiments and rheological measurements also confirmed a border between the dilute and the transition regimes located around 150 g/L at 7 °C and around 160–170 g/L at 20 °C.

These results can be explained considering the increase in hydration and voluminosity of the casein micelles with the temperature decrease at 7 °C.

The lower sol–gel transition concentration determined at 7 °C compared with 20 °C can be explained by the fact that a particle of higher voluminosity leads to a sol–gel transition regime at lower concentration. When the voluminosity is taken into account, a unique effective volume fraction of 0.71 is found for the sol–gel transition at 7 °C and 20 °C (Figure 10b). This result is consistent with the fact that the transition is a jamming transition, ruled by the crowding of the constituent particles and occurring at a same volume fraction for particles of similar polydispersity. Furthermore, the fact that casein micelles are more hydrated and have a higher voluminosity at 7 °C than at 20 °C is consistent with casein micelles that are less compressible at 7 °C compared with 20 °C since osmotic pressure is the cost to remove water [14].

Moreover, the difference of rheological behaviors of casein micelles between 7 °C and 20 °C is consistent with voluminosity changes of casein micelles at 7 °C compared with 20 °C. In the transition regime, casein micelle dispersions at 7 °C and 20 °C exhibited a shear-thinning behavior, with shear-thinning indexes at 7 °C and 20 °C having very similar values when voluminosity is taken into account (Figure 7b).

In the concentrated regime, most of the water that separates casein micelles has been removed and compression constrains casein micelles to deform and de-swell [14]. Therefore, in this regime, the applied osmotic pressure corresponds to the compression resistance of casein micelle core that is consistent with an increase in water content in the micellar core (native hydration) due to a decrease in temperature. This increase in native hydration of the core is consistent with the fact that the casein micelle has more affinity with water at 7 °C than at 20 °C, and this is why it resists more to its own compression in this last regime.

## 5. Conclusions

In this work, we have studied the properties of concentrated casein micelles at 7 °C and 20 °C by measuring the osmotic pressure, the sol–gel transition, and the rheological properties of dense dispersions. Osmotic stress technique is a convenient and nondestructive method to make dense casein micelle dispersions. Furthermore, understanding the behavior of casein micelles at a concentration higher than close packing is fully relevant for all concentration processes, and especially filtration.

Regardless of the temperature, casein micelle dispersions followed the typical three compression regimes with first, a dilute regime where casein micelles are not in contact, then a sol–gel transition regime where casein micelles begin to interact, and finally a concentrated regime where casein micelles behave as soft solids. The remarkable result of the current work is that casein micelles were less easy to compress at 7 °C compared with 20 °C.

Identically, regardless of the temperature, dense casein micelle dispersions followed successive rheological behaviors with the rise of concentration. First casein micelle dispersions behave quite as Newtonian fluids, then they exhibit a shear-thinning behavior and a yield stress, and finally a sol–gel transition. The remarkable result of this work is that the changes in shear-thinning behavior, appearance of yield stress, and sol–gel transition were observed at lower concentrations and with stronger intensity at 7 °C compared with 20 °C.

Moreover, this work has pointed out that these differences of compressibility and sol–gel transition concentration observed between 7 °C and 20 °C were fully consistent with a release of soluble β-casein and nanoclusters CaP and an increased casein micelle hydration and apparent voluminosity at 7 °C compared with 20 °C. Therefore, changes in casein micelle structure at 7 °C and 20 °C were responsible for the lower compressibility and lower sol–gel transition concentration at 7 °C compared with 20 °C.

However, investigations are still needed to support these last conclusions and attempt to quantify and explain the differences of compressibility at 7 °C and 20 °C, such as a modeling approach.

With this work, we bring new and important information on the way casein micelles behave and gel at 7 °C and 20 °C in concentrated systems. This information may be relevant to understand the behavior of casein micelle deposited at the membrane surface during ultrafiltration or microfiltration of milk. Casein micelles are less compressible and gel at a lower concentration at 7 °C compared with 20 °C, suggesting that casein micelles deposit formed at the membrane surface at 7 °C would be more gelled, more concentrated, and thicker than at 20 °C. Therefore, if milk is assimilated to concentrated casein micelle dispersions, conclusions of this work show that milk filtration at ambient temperature could be preferred since casein micelle deposit formed during milk filtration would be less cohesive at 20 °C than at 7 °C. Indeed, operating milk filtration at ambient temperature would allow to form deposit that would be easier to remove, for instance during rinsing operations.

## Figures and Tables

**Table 1:** Composition of casein isolate powder. Average values of relative concentration are given in wt. % of total solids content (% TS) with a standard deviation of ± 0.2 wt. %.

Total Solids (TS) (wt. %)	Total Nitrogen Matter (% TS)	Casein (% TS)	Minerals (% TS)	Non-Casein Nitrogen Matter (NCN) (% TS)	Serum Proteins (% TS)	Non-Protein Nitrogen Matter (% TS)
94.5	83.9	75.9	8.4	8.0	7.2	0.8

## Supplementary Materials

The supplementary materials are available online at https://www.mdpi.com/2304-8158/8/12/652/s1.
