# Supplementary material for: Major Role of Voluminosity in the Compressibility and Sol–Gel Transition of Casein Micelle Dispersions Concentrated at 7 °C and 20 °C
Source: Foods. 2019 Dec 6;8(12):652. doi: 10.3390/foods8120652 (PMC6963684; doi:10.3390/foods8120652)
Supplement: Supplementary file 1 [file foods-08-00652-s001.pdf]

Supplementary materials

S.1. PEG/water osmotic pressure data plotted in Figure 1

The following data are reported verbatim from the website:

https://scholars.huji.ac.il/danielharries/book/peg-20k

Data were measured in the laboratory of R. Peter Rand, Department of Biological Sciences, Brock University, St. Catharines, Ontario, Canada. All measurements reported here were done at 20 °C except when it is noticed.

The molecular weights are nominal.

polyethylene glycol MW 20,000

wt%	log P (dynes/cm^2)
1.5	4.59
2.9	4.98
3.6	5.15
4.7	5.34
5.8	5.55
6.3	5.63
7.1	5.76
9.0	5.89
12.1	6.08
13.1	6.18
14.2	6.29
16.0	6.47
18.3	6.70
18.8	6.68
20.8	6.77
21.6	6.83
22.2	6.83
22.8	6.94
19.0	6.68
22.1	6.83
23.7	6.93
25.0	6.94
26.2	7.03
28.8	7.11
32.7	7.26
34.8	7.34
35.6	7.32
38.0	7.45
39.5	7.48
41.7	7.54

43.2	7.59
45.5	7.67
47.5	7.73

polyethylene glycol MW 20,000

At a Temperature of 7-8 degrees C

wt%	log P (dynes/cm^2)
15.3	6.56
17.8	6.75
18.3	6.79
19.5	6.82
20.8	6.91
21.3	6.92
22.9	6.99
23.2	7.00
24.3	7.04

S.2. Casein concentration equation (eq. 4)

The detailed process of determination of casein concentration equation (eq. 4) was taken as the gram of casein per gram of dispersion:

$$[\mathrm{Cas}]_{\mathrm{g/g}} = \frac{\text{casein content in dialysis bag (g)}}{m_{disp}\ (\mathrm{g})}$$

$$[\mathrm{Cas}]_{\mathrm{g/g}} = \frac{\text{casein proportion in dialysis bag } \times m_{dry\ matter\ of\ dialysis\ bag}}{m_{disp}}$$

$$[\mathrm{Cas}]_{\mathrm{g/g}} = \frac{(1 - \mathrm{NCN}) \times TS_{powder} \times (TS_{disp} - TS_{UF})}{1 - TS_{UF}}$$

where m_{disp} was casein dispersion mass, $m_{dry\ matter\ of\ dialysis\ bag}$ was mass of dry matter in the dialysis bag, TS_{powder}, TS_{disp}, TS_{UF} were respetively total solids in powder, dispersion and UF permeate.

For the derivation, it is assumed that only casein and non casein nitrogen were in the powder and were present in the dialysis bag. We suppose that other compounds (low molecular weight coumpounds) went through the dialysis bag and we neglected presence of fat globules which were present in very small proportion.

S.3. Determination of sol-gel transition with change of elastic modulus slope

Figure S.3.1 shows another method to determine sol-gel transition of casein micelle dispersions. Slopes of elastic modulus were represented at 7 and 20 °C at different concentrations and sol-gel transition is defined as the concentration where elastic modulus slope increase strongly. For better readability, loss modulus was not presented but the curves were identical. With this method, values very similar to the ones obtained by another method (Figure 10) were found: a sol-gel transition of around 155 - 160 g/L at 7 °C and of around 170 – 175 g/L at 20 °C.

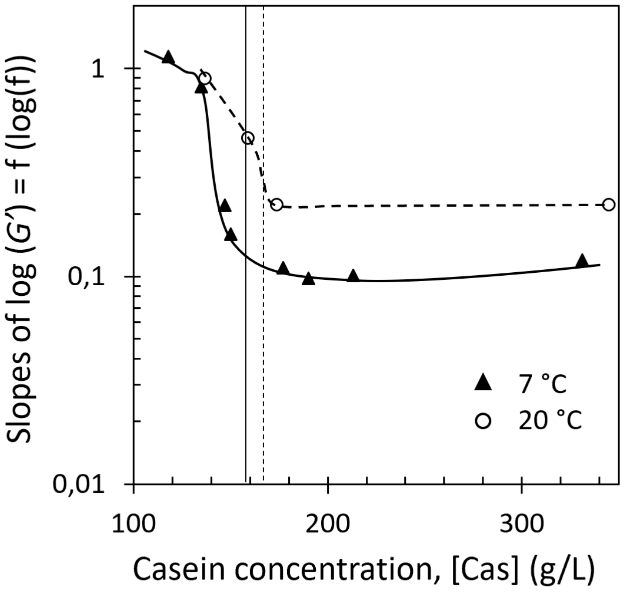

Figure S.3.1. The slopes of elastic G' modulus over frequency of casein micelle dispersions in UF permeate at equilibrium conditioning temperature of 7 °C (solid triangles) and at 20 °C (open circles) as a function of casein concentration. Vertical lines correspond to sol-gel transition concentration of [Cas] = 155 - 160 g/L at 7 °C (solid line) and [Cas] = 170 - 175 g/L at 20 °C (dashed line).
